# miR-218-5p inhibits the stem cell properties and invasive ability of the A2B5+CD133- subgroup of human glioma stem cells

**DOI:** 10.3892/or.2022.8383

**Published:** 2022-08-01

**Authors:** Zhiwu Wu, Yong Han, Yanyan Li, Xuetao Li, Ting Sun, Guilin Chen, Yulun Huang, Youxin Zhou, Ziwei Du

Oncol Rep 35: 869–877, 2016; DOI: 10.3892/or.2015.4418

Following the publication of this paper, it was drawn to the Editors' attention by a concerned reader that Fig. 5 on p. 874 contained a series of DAPI panels within the figure that looked unexpectedly similar in appearance, with the similarities also evident in the second and ‘Merge’ data columns; moreover, of especial note, the similarities in the ‘DAPI’ panels for the Blank control experiments shown in Fig. 5C and D only affected a partial section of the data. In addition, the ‘blank’ and ‘miR-control’ panels in Fig. 6A also appeared to contain overlapping data.

Independently of the issues that were raised by the interested reader, the authors themselves requested that their paper be retracted on account of having identified some problems with the presentation of various of the figures, and no longer being able to access their original data. The Editor of *Oncology Reports* has agreed that this paper should be retracted from the Journal, and apologizes to the readership for any inconvenience caused.

